# Supplementary material for: Using deep learning to identify inherited retinal diseases based on wide-field retinal imaging data
Source: PLoS One. 2026 May 11;21(5):e0348866. doi: 10.1371/journal.pone.0348866 (PMC13160341; doi:10.1371/journal.pone.0348866)
Supplement: S2 Table — (DOCX) [file pone.0348866.s002.docx]

**S2 Table:** Hyperparameters used for model training in all of experiments

| **Hyperparameters** | |
| --- | --- |
| Number of iterations | 100 |
| Batch size | 36 |
| **Augmentations** |  |
| Augmentations | Vertical flipping, Horizontal flipping, Rotation, Translation |
| Probability of applying each augmentation | 0.3 |
| **Optimization** |  |
| Optimizer | Adam |
| Learning Rate | 10^-3^ |
| Adam optimizer momentum parameters (Betas) | 0.80, 0.98 |
| **Loss function** | Weighted Cross Entropy |
| Weights | $W\left( I \right)=\frac{1}{\frac{count(I)}{\sum_{j\in train data} count(j)}}$  $I$: iterate over all classes in the classification task  $W(I):$ is the weight assigned to the class $I$  $count(I):$ represents the number of occurrences of class  $I$in the dataset.  $\sum_{j\in train data} count(j)$**:** the sum of occurrences across all classes. |

**Weighted cross-entropy**

To address the class imbalance and achieve a balance between improving performance with minority classes while preventing bias toward majority classes, we used a weighted cross-entropy loss function was employed; the class weights were calculated using the formula and parameter values listed in Table S1. This approach assigns a weight that is inversely proportional to the class frequency ratio, ensuring that less frequent classes are weighted more heavily while frequent classes are weighted less heavily. The class weights were computed separately for each study based on the class distributions in the training dataset. By incorporating these weights into the cross-entropy loss calculation, the model's sensitivity for minority classes was increased while maintaining a balanced overall performance.

**Partition**

When partitioned into different sets, the intra-patient structure remains within each set, meaning that all images obtained from a single patient (including both eyes from all visits, where applicable) were grouped together in the same dataset.
