# Supplementary material for: Using deep learning to identify inherited retinal diseases based on wide-field retinal imaging data
Source: PLoS One. 2026 May 11;21(5):e0348866. doi: 10.1371/journal.pone.0348866 (PMC13160341; doi:10.1371/journal.pone.0348866)
Supplement: Attachment — Submitted filename: Response to reviewers-13-7-2025.docx [file pone.0348866.s003.docx]

Journal Requirements:

1. Please ensure that your manuscript meets PLOS ONE's style requirements, including those for file naming. The PLOS ONE style templates can be found at

<https://journals.plos.org/plosone/s/file?id=wjVg/PLOSOne_formatting_sample_main_body.pdf> and

[https://journals.plos.org/plosone/s/file?id=ba62/PLOSOne_formatting_sample_title_authors_affiliations.pdf](https://track.editorialmanager.com/CL0/https:%2F%2Fjournals.plos.org%2Fplosone%2Fs%2Ffile%3Fid=ba62%2FPLOSOne_formatting_sample_title_authors_affiliations.pdf/1/010f019782879b5c-c6c87947-925f-4175-9f26-18ef2f88339e-000000/WF9ZcBx4ce3ZvbjRO855X5w-hqOSDNXFHHvlsWKrFdE=216)

We have revised the manuscript according to PLOS ONE's style requirements.

2. Please note that PLOS ONE has specific guidelines on code sharing for submissions in which author-generated code underpins the findings in the manuscript. In these cases, we expect all author-generated code to be made available without restrictions upon publication of the work. Please review our guidelines at <https://journals.plos.org/plosone/s/materials-and-software-sharing#loc-sharing-code> and ensure that your code is shared in a way that follows best practice and facilitates reproducibility and reuse.

We added, line 199: Upon acceptance of the manuscript, the tool will be uploaded to a public repository and made freely available for use.

3. When completing the data availability statement of the submission form, you indicated that you will make your data available on acceptance. We strongly recommend all authors decide on a data sharing plan before acceptance, as the process can be lengthy and hold up publication timelines. Please note that, though access restrictions are acceptable now, your entire data will need to be made freely accessible if your manuscript is accepted for publication. This policy applies to all data except where public deposition would breach compliance with the protocol approved by your research ethics board. If you are unable to adhere to our open data policy, please kindly revise your statement to explain your reasoning and we will seek the editor's input on an exemption. Please be assured that, once you have provided your new statement, the assessment of your exemption will not hold up the peer review process.

We definitely agree. We provided all data. We changed the data availability statement of the submission form.

Line 119: All data supporting the findings of this study are available within the manuscript and in supplementary materials.

We note that you have included the phrase “data not shown” in your manuscript. Unfortunately, this does not meet our data sharing requirements. PLOS does not permit references to inaccessible data. We require that authors provide all relevant data within the paper, Supporting Information files, or in an acceptable, public repository. Please add a citation to support this phrase or upload the data that corresponds with these findings to a stable repository (such as Figshare or Dryad) and provide and URLs, DOIs, or accession numbers that may be used to access these data. Or, if the data are not a core part of the research being presented in your study, we ask that you remove the phrase that refers to these data.

As above, all data supporting the findings of this study are available within the manuscript and in supplementary materials.

5. [Please remove all personal information, ensure that the data shared are in accordance with participant consent, and re-upload a fully anonymized data set.

Note: spreadsheet columns with personal information must be removed and not hidden as all hidden columns will appear in the published file.

Additional guidance on preparing raw data for publication can be found in our Data Policy (https://journals.plos.org/plosone/s/data-availability#loc-human-research-participant-data-and-other-sensitive-data) and in the following article: http://www.bmj.com/content/340/bmj.c181.long.](http://track.editorialmanager.com/CL0/http:%2F%2Fwww.bmj.com%2Fcontent%2F340%2Fbmj.c181.long./1/010f019782879b5c-c6c87947-925f-4175-9f26-18ef2f88339e-000000/snPCnuLZYf0yc-uXHSaWLKh4X5SEYqBRJV3zQ5JERtg=216)

 A table with patients' information is provided as supplementary material.

**Additional Editor Comments:**

1. This manuscript is significantly weakened by the fact that the genetic defects considered applies to a limited population in which the research was conducted. Findings are therefore not widely generalizeable. The authors need to discuss this in more detail, though it was mentioned as a weakness of the study in the last section of the discussion.

We agree that the genetic spectrum evaluated in this study reflects the most common IRD-causing genes in our local Israeli population and does not encompass the full diversity of mutations observed globally. This was primarily due to the need for sufficient sample sizes for training robust classification models. We now expand on this limitation in the Limitations paragraph:

Line 322: Third, we focused on only 25 of the more than 300 known IRD-associated genes, selected based on their prevalence in the Israeli population. This may limit the model’s applicability to populations with different genetic backgrounds, suggesting the need for validation in larger, more genetically diverse, and multi-center cohorts.

And also in Discussion:

Line 311: While our findings demonstrate strong diagnostic performance within the studied population, they must be interpreted in the context of the genetic background on which the models were trained. The gene panel used reflects the most prevalent IRD-associated mutations in the Israeli population, and although this approach enabled robust classification within the cohort, it may not capture the full genetic and phenotypic variability seen in other populations. Therefore, the clinical applicability of our models to more diverse or globally representative cohorts remains to be determined.

2. I also suggest that the introduction be made more concise and straight to the point, highlighting the need for this research and potential of the findings. This is not clearly mentioned in the introduction
We appreciate the suggestion to make the introduction more concise and focused. We have revised the introduction (see pages 1–2) to streamline the background and more directly highlight the motivation and significance of this study. Specifically, we now clearly state:

Line 95: Despite the increasing availability of genetic testing, access remains limited in many regions due to cost, infrastructure, or lack of awareness. An accurate, image-based tool that can assist in the early identification of IRDs—prior to or when genetic testing is not readily available—could substantially reduce diagnostic delays and support timely clinical decision-making. This study addresses a critical unmet need by evaluating a novel deep learning–based method for classifying IRD versus non-IRD using multimodal wide-field retinal images and investigating the potential to predict causative gene groups directly from imaging data. Such an approach may be especially valuable in resource-limited settings and can help prioritize patients for confirmatory genetic workup.

3. The other important issue, is that several inherited disease including RP and Stargardts disease phenotypes can be identified with clinical examination. Therefore, one wonders the utility of this model. Can the authors provide supporting evidence on the clinical utility of this model.

We thank the reviewer for this insightful comment. We agree that clinical examination, including multimodal imaging, can often identify characteristic phenotypes such as retinitis pigmentosa (RP) or Stargardt disease. However, many IRDs present with atypical features or show phenotypic overlap, particularly in early stages, making diagnosis challenging—even for experienced clinicians. In addition, in non-specialist or underserved settings, access to retinal experts and genetic testing may be limited. In such cases, our model can provide valuable support by suggesting a likely diagnosis based on imaging alone, guiding timely referrals and focused genetic workup. Moreover, deep learning offers a reproducible, objective tool that complements clinical expertise and may help standardize diagnostic approaches across diverse healthcare environments.

We have revised the Discussion section to highlight these points more explicitly

Line 300: Although certain IRDs, such as RP and Stargardt disease, can often be recognized through clinical examination and multimodal imaging by experienced ophthalmologists, diagnosis remains challenging in many real-world scenarios. Atypical phenotypes, early-stage disease, or overlapping features with non-IRD conditions may obscure the diagnosis. Moreover, in non-specialist or resource-limited settings where access to IRD experts and comprehensive testing is limited, deep learning–based tools can provide valuable decision support. Such models can help general ophthalmologists detect subtle or under-recognized IRD features, enabling earlier referral and targeted genetic testing. Furthermore, the objective and reproducible nature of deep learning–based classification offers a standardized approach to initial IRD screening that is independent of examiner expertise, potentially improving diagnostic equity across healthcare settings.

**Reviewers' comments:**

**Reviewer #1:**

The research paper has a very shallow introduction.

We appreciate the reviewer’s comment regarding the depth of the introduction.

In response, we have rewritten the Introduction section to make it more concise, focused, and informative. We now more clearly outline the clinical burden of inherited retinal dystrophies (IRDs), the limitations of current diagnostic pathways, and the unmet need for accessible, automated diagnostic tools. Additionally, we emphasize the rationale and potential clinical utility of our deep learning–based approach and how it builds on and differs from previous work. These revisions aim to provide a stronger motivation for the study and better contextualize our research within the broader field.

The methodology of the research is not clear.

The Methods section has been thoroughly revised to improve clarity, structure, and transparency, with detailed descriptions of the dataset, classification tasks, model architecture, and evaluation procedures

The information about dataset is mentioned clearly.

We thank the reviewer for noting that the dataset description is clear.

The technical discussion of results is very vague.

We thank the reviewer for the comment.

The Discussion section has been thoroughly revised to improve clarity, focus, and technical depth. We have better contextualized our findings within the existing literature, clarified the clinical implications, and expanded the discussion of limitations and future directions.

The optimization of hyperparameters has not been discussed.

We thank the reviewer for this important observation. We have now added a detailed description of the hyperparameter optimization process to the Methods section and supplementary material

Line 178: Hyperparameters—including learning rate, augmentation probabilities, cropping range, and regularization parameters—were selected based on five-fold cross-validation, and final values were chosen to optimize validation performance and generalization

**Reviewer #2:**

In this study, a multimodal retinal image analysis method based on deep learning is proposed to automatically identify hereditary retinal diseases (IRD) and related pathogenic genes. The research results show high classification performance (for example, IRD vs non-IRD classification accuracy reaches 0.95). However, there is room for improvement in data diversity, model external verifiability, clinical interpretation and research details. The following are specific opinions:

1. The data set used in this study mainly comes from a single medical center in Israel, and only contains 25 most common IRDs-related genes. Although this is representative in the local area, it may limit the global applicability of the model. If possible, it is suggested to supplement the data of other regions or populations to verify the diagnostic performance of the model.

We thank the reviewer for this important comment. We acknowledge that our dataset, derived from a single medical center in Israel and focusing on the 25 most common IRD-related genes in this population, may limit the global generalizability of the model. Unfortunately, at this time, we do not have access to additional datasets from other regions or populations to externally validate the model.

In Limitations, Line 322: Third, we focused on only 25 of the more than 300 known IRD-associated genes, selected based on their prevalence in the Israeli population. This may limit the model’s applicability to populations with different genetic backgrounds, suggesting the need for validation in larger, more genetically diverse, and multi-center cohorts.

2. The study did not elaborate on the interpretability of AI model. It is suggested to increase the analysis and explanation of AI model decision to help clinicians understand its diagnostic logic, thus enhancing the credibility in practical application.

We thank the reviewer for highlighting the importance of model interpretability. We agree that understanding the decision-making process of AI models is crucial for clinical acceptance. Although our current study does not include an analysis of interpretability techniques such as saliency maps or Grad-CAM, we recognize their potential value. Due to resource limitations and the current scope of the project, we were unable to incorporate these explainability methods at this time. However, we emphasize that our models are intended as diagnostic support tools to complement, not replace, clinical judgment. Future research will aim to include interpretability analyses as part of ongoing efforts to enhance clinical trust and applicability.

Line 342: Finally, while this study demonstrates high diagnostic accuracy of our deep learning models, it currently does not incorporate interpretability techniques such as saliency maps or Grad-CAM to explain the basis of model predictions. We acknowledge that such explainability tools are important to increase clinician trust and facilitate integration into practice. Due to resource constraints, these analyses were beyond the scope of this study but are planned for future work.

3. The paper mentioned the image preprocessing and enhancement methods, but did not provide specific parameters. It is suggested to supplement the technical details, including the concrete realization of data enhancement and the process of super-parameter tuning.

We agree with your comment.

We have now added a detailed description of the hyperparameter optimization process to the Methods section and supplementary material

Line 178: Hyperparameters—including learning rate, augmentation probabilities, cropping range, and regularization parameters—were selected based on five-fold cross-validation, and final values were chosen to optimize validation performance and generalization

4. This study did not fully explore the feasibility of the model in resource-poor and backward areas (such as relying on high-end imaging equipment Optos system). It is suggested to increase discussion on this limitation and put forward solutions.

We thank the reviewer for highlighting the important issue of applicability in resource-poor settings. Indeed, our study utilized wide-field imaging systems such as Optos, which may not be readily available in all clinical environments, particularly in under-resourced or rural areas. We have now expanded the Discussion section to address this limitation explicitly.

Line 333: The study relied on wide-field imaging systems, such as the Optos platform, which may not be available in many resource-poor or rural healthcare settings. This dependence on high-end equipment could restrict the immediate applicability of our model in such environments. To enhance accessibility, future work could focus on adapting the deep learning models to utilize images from more commonly available, lower-cost fundus cameras with narrower fields of view. Additionally, the development of AI tools compatible with portable, affordable imaging devices may facilitate broader implementation in under-resourced areas. Validation studies in these settings will be essential to assess model performance and to tailor solutions that promote equitable access to IRD diagnostics worldwide.

5. For the evaluation of research results, please use medical indicators. You can refer to this paper(Guidelines on clinical research evaluation of artificial intelligence in ophthalmology (2023). Int J Ophthalmol 2023;16(9):1361-1372
DOI:10.18240/ijo.2023.09.02).

Thank you for the valuable suggestion. We appreciate the reference to the recent guidelines on clinical research evaluation of AI in ophthalmology. The reference was included #31.

We added, line 204: In our analysis, we adopted a hybrid evaluation strategy that integrates metrics commonly reported in artificial intelligence and machine learning research (e.g., accuracy, precision / positive predictive value, F1 score, area under the curve (AUC)) with elements that align with the expectations of clinical and medical journals, such as class-wise accuracy [31]. Calibration measures and decision curve analysis were not performed at this stage, as the current analysis did not extend to clinical deployment scenarios. Similarly, comparison with human experts was outside the scope of this phase but remains an important consideration for future validation.
