# Supplementary material for: Using deep learning to identify inherited retinal diseases based on wide-field retinal imaging data
Source: PLoS One. 2026 May 11;21(5):e0348866. doi: 10.1371/journal.pone.0348866 (PMC13160341; doi:10.1371/journal.pone.0348866)
Supplement: Attachment — Submitted filename: Response to reviewers-13-12-2025.docx [file pone.0348866.s004.docx]

**Comments to the Author**

1. If the authors have adequately addressed your comments raised in a previous round of review and you feel that this manuscript is now acceptable for publication, you may indicate that here to bypass the “Comments to the Author” section, enter your conflict-of-interest statement in the “Confidential to Editor” section, and submit your "Accept" recommendation.

Reviewer #2: (No Response)

Reviewer #3: (No Response)

2. Is the manuscript technically sound, and do the data support the conclusions?

The manuscript must describe a technically sound piece of scientific research with data that supports the conclusions. Experiments must have been conducted rigorously, with appropriate controls, replication, and sample sizes. The conclusions must be drawn appropriately based on the data presented.

Reviewer #2: (No Response)

Reviewer #3: No

We thank the reviewer for raising concerns regarding technical rigor and data support. We acknowledge that our study has certain limitations, including a relatively small sample size for some IRD-associated genes, the use of gene groups (Group 1 and Group 2) primarily to ensure sufficient sample sizes for proof-of-concept demonstration, the lack of external validation, and restriction to a single-center cohort.

However, we have mitigated these limitations by using careful patient-level partitioning, targeted data augmentation, and robust cross-validation to prevent overfitting. Despite these constraints, our data consistently demonstrate high accuracy for both IRD vs. non-IRD classification and gene-level prediction within the studied cohort. We have revised the Discussion to explicitly state these limitations and to clarify that our conclusions pertain to proof-of-concept performance in a genetically characterized cohort, with generalizability to larger or more diverse populations requiring further validation.

3. Has the statistical analysis been performed appropriately and rigorously?

Reviewer #2: (No Response)

Reviewer #3: N/A

4. Have the authors made all data underlying the findings in their manuscript fully available?

The [PLOS Data policy](http://track.editorialmanager.com/CL0/http:%2F%2Fwww.plosone.org%2Fstatic%2Fpolicies.action%23sharing/1/010f019ad8783c4d-151e0ecc-7a53-42fd-8367-0709ee93e1db-000000/G63WfLxzUTtm5umSAc_f-jfCv892RSboyvYESRjRzeg=239) requires authors to make all data underlying the findings described in their manuscript fully available without restriction, with rare exception (please refer to the Data Availability Statement in the manuscript PDF file). The data should be provided as part of the manuscript or its supporting information, or deposited to a public repository. For example, in addition to summary statistics, the data points behind means, medians and variance measures should be available. If there are restrictions on publicly sharing data—e.g., participant privacy or use of data from a third party—those must be specified.

Reviewer #2: (No Response)

Reviewer #3: Yes

5. Is the manuscript presented in an intelligible fashion and written in standard English?

PLOS ONE does not copyedit accepted manuscripts, so the language in submitted articles must be clear, correct, and unambiguous. Any typographical or grammatical errors should be corrected at revision, so please note any specific errors here.

Reviewer #2: (No Response)

Reviewer #3: Yes

**6. Review Comments to the Author**

Please use the space provided to explain your answers to the questions above. You may also include additional comments for the author, including concerns about dual publication, research ethics, or publication ethics. (Please upload your review as an attachment if it exceeds 20,000 characters)

Reviewer #2: (No Response)

Reviewer #3: This manuscript investigates the application of deep learning to identify IRDs from wide field retinal images. The methodology used has been previously reported and is sound, however, there are a number of limitations in this manuscript that need to be addressed:

**Major**

1. How was the limited availability of data addressed in the training and subsequent testing of the CNNs? They have mentioned that data is limited but there is no specific mention about how this was managed and were images from training and testing stratified by patients or images as each patient probably had more than one set of retinal images included.

Thank you for this important comment. Although our dataset included multiple retinal images per patient, the manuscript now clarifies how we handled limited data, how the CNNs were trained and evaluated, and how we prevented patient-level data leakage.

To address limited data availability, we applied several measures:

1) Patient-level stratification: All images from the same patient were assigned exclusively to either the training, validation, or testing set. This prevented information leakage and ensured a realistic evaluation of model performance.
2) Data augmentation: We used standard augmentation techniques (horizontal/vertical flips, rotation, brightness and contrast jitter) to increase dataset variability and reduce overfitting.
3) Cross-validation: We performed five-fold cross-validation using patient-level partitions, further improving robustness under limited sample size.

These clarifications have now been added to the Methods section (Dataset and Model Training) and briefly referenced in the Discussion.

Line 170: To prevent data leakage arising from multiple images belonging to the same patient, all dataset partitions were performed at the patient level. Thus, all images from a given patient were assigned exclusively to the training, validation, or testing set. This ensured that the CNNs were evaluated on entirely unseen patients.

Line 178: In addition, to mitigate the impact of limited sample size, we applied standard data-augmentation techniques as random vertical and horizontal flips, as well as translations of up to 15 pixels in both directions.

Line 187: Hyperparameters, including learning rate, augmentation probabilities, cropping range, and regularization parameters, were selected based on five-fold cross-validation, and final values were chosen to optimize validation performance and generalization. Each fold maintained strict patient-level separation across training and testing subsets.

Limitations, line 347: First, although the sample size for both IRD and non-IRD groups was relatively small compared to some previous studies, our dataset is unique and of high quality, and careful patient-level partitioning together with targeted augmentation strategies helped reduce overfitting and ensured that the reported performance reflects true generalization rather than memorization of images from the same individuals.

2. How common are the 25 genetic causes of IRDs that were selected in the study population? This will help the reader try to understand the potential local relevance of this work in the studied group.

We thank the reviewer for this important point. In our cohort, the 25 causative genes selected for model development account for approximately 63% of all IRD cases evaluated at our center during the study period. We have now added this information explicitly in the Methods section to clarify the local relevance and representativeness of the selected genes.

Line 127: These 25 genes accounted for approximately 63% of all genetically confirmed IRD cases evaluated at our center during the study period, highlighting their substantial local relevance and their suitability for inclusion in the model.

And in Limitations, line 357: The 25 causative genes included in our analysis accounted for approximately 63% of all IRD cases in our cohort, indicating that the gene selection reflects the most prevalent local etiologies; however, rarer genetic subtypes were not represented.

3. The authors reference some other work in the literature and specifically mention Eye2Gene but they have not included the groups most recent published work e.g., <https://doi.org/10.1038/s42256-025-01040-8>

We thank the reviewer for this important observation. We apologize for having missed this reference, as it was published shortly before our submission. We agree that inclusion of the most recent Eye2Gene publication is necessary to provide a complete and up-to-date comparison with existing deep learning approaches for IRD. We have now added this citation (#34) to the Discussion and integrated a sentence summarizing the study’s methods and findings, as well as its relevance to our work.

Specifically, we note that the new Eye2Gene article further expanded their multimodal framework but continued to report modest gene-level accuracy despite the considerably larger dataset used for training. This updated comparison helps contextualize our study’s performance and emphasizes the strengths of our approach.

Line 306: In addition, a more recent publication from the Eye2Gene group [34], based on 58,030 scans from 2,451 patients, introduced an updated multimodal deep learning framework integrating FAF, color fundus imaging, and clinical metadata. Despite this substantially larger and more heterogeneous dataset, the model achieved only moderate gene-level performance, with a top-5 accuracy of approximately 76%, which remains lower than the accuracies reported in our study, despite leveraging a significantly larger and more heterogeneous dataset.

Line 314: The recent Eye2Gene model also included IRD versus non-IRD classification, achieving a receiver operating characteristic of 0.98 [34]. Our model achieved a mean accuracy of 0.94, demonstrating that, even with far fewer images, our targeted approach delivers comparable performance to larger-scale generic models in both accuracy and clinical relevance.

4. There is no clear indication from the figures provided how difficult the classification task is for the model. Figure 1 images are not labelled as to the diagnosis and are not clear. For the IRD/non-IRD task as most of the images are normal and many of the non-IRD cases won’t be expected to have changes on FAF. This also could explain the strength of the FAF model alone compared to the combined model. Line 248-251 follows on from this point, how can you make that assumption (that the model is better than an ophthalmologist/retinal specialist) when you have not made that comparison with your dataset.

We thank the reviewer for this important comment. We agree that the classification task and comparison to human experts were not clearly described. **We have revised the Discussion to clarify that many non-IRD cases in our dataset exhibit normal-appearing FAF images, which may simplify the binary IRD vs. non-IRD classification task. Accordingly, while our model demonstrates high performance, we have removed the direct comparison to ophthalmologists or retinal specialists, as no such evaluation was performed on this dataset.**

**We also acknowledge that Figure 1 was intended to illustrate representative image diversity rather than to convey diagnostic difficulty, and we have added clarifying statements in the figure legend to reflect this.** These revisions provide a more accurate interpretation of our results and better contextualize the model’s performance.

We also note that the original Figure 1 images are of substantially higher resolution and sharpness than may appear in the automatically generated PDF version, due to document conversion and compression.

Line 260: We note that many non-IRD cases in our dataset exhibit normal-appearing FAF images, which may simplify the binary classification task. Therefore, while performance metrics are high, caution is warranted in interpreting these results as surpassing human experts, as we did not perform a direct comparison with ophthalmologists or retinal specialists on this dataset.

Line 321: While we did not perform a direct comparison with human experts in our dataset, our model produces consistent, reproducible predictions based on standardized image inputs, which could support earlier detection and referral in real-world settings.

Line 510: Figure 1: Representative fundus autofluorescence (FAF, left) and pseudo color (pCF, right) images illustrating variability in the dataset, including differences in intensity, magnification, text annotations, and partial occlusion by eyelashes or eyelids. These images are intended to show the diversity of imaging conditions rather than the difficulty of classification or the underlying diagnosis.

**Minor**

1. The CF images used are not true colour but pseudo colour optos images. Please consider changing CF throughout to reflect this.

We thank the reviewer for this important observation. You are correct that the Optos “color fundus” images are pseudo-color rather than true color. We have carefully revised the manuscript to replace all instances of “color fundus (CF)” with “pseudo-color fundus (pCF)” to accurately reflect the imaging modality used. This change has been applied throughout the text, figures, and figure legends.

2. What was the rational for deciding on Group 1 and Group 2? You note that this is arbitrary and does not include all of the 25 genes that are used in experiment 1. This does not link in to lines 450-451 where you suggest gene-level predictions would be useful to guide testing but have not been able to demonstrate that in your work. Clarifying the message and rational here would be helpful.

We thank the reviewer for this insightful comment. The selection of Group 1 and Group 2 genes was indeed based on pragmatic considerations rather than biological clustering. Specifically, we aimed to maximize the robustness of the deep learning models given the limited number of patients for certain genes. Some genes had very few cases (e.g., RPE65, n=5), which would have prevented meaningful model training if treated individually. Therefore, we grouped genes empirically based on prevalence in our cohort and preliminary model performance, prioritizing balanced sample sizes and sufficient representation for training.

We acknowledge that these groups do not include all 25 genes used in Experiment 1 and that gene-level prediction across all genes remains an aspirational goal. The current grouping allowed us to demonstrate proof-of-concept for gene-level prediction, while avoiding overfitting due to sparse data. We have clarified this point in the manuscript to make the rationale explicit and consistent with the discussion of gene-level predictions.

Line 162: Groups were defined empirically to balance sample size across classes and optimize model performance in preliminary experiments, rather than based on biological or clinical similarity. This approach allowed us to explore gene-level prediction in a proof-of-concept framework while avoiding overfitting due to sparse data.

Line 280: We note that the gene groups (Group 1 and Group 2) were selected primarily to ensure sufficient sample sizes for robust model training rather than to reflect biological or clinical relationships. While these groups do not include all 25 genes used in Experiment 1, they enable proof-of-concept demonstration of gene-level prediction from imaging data. Future work with larger cohorts will be needed to extend this approach to additional genes and to fully realize the potential for imaging-guided genetic testing.

3. Line 292 - how is that an advantage of your approach, surely that is the case for any deep learning approach to this problem?

While it is true that most deep learning models are independent of clinician expertise, our approach demonstrates a specific advantage because it achieves high accuracy even with a relatively small dataset. This means that in real-world clinical settings, especially in regions or centers with limited access to IRD specialists, our model can provide reliable, reproducible predictions where larger generic models may struggle due to heterogeneity or low representation of specific gene phenotypes. Furthermore, the multimodal design (pCF + FAF) enhances robustness and accuracy, which is not guaranteed for all deep learning approaches.

We added in Discussion: Line 324: While many deep learning models are inherently independent of clinician input, our targeted, multimodal approach achieves high accuracy even with a relatively small cohort. This makes it particularly advantageous in clinical contexts with limited IRD expertise or heterogeneous patient populations, where standard deep learning models might underperform.
