# Supplementary material for: Using deep learning to identify inherited retinal diseases based on wide-field retinal imaging data
Source: PLoS One. 2026 May 11;21(5):e0348866. doi: 10.1371/journal.pone.0348866 (PMC13160341; doi:10.1371/journal.pone.0348866)
Supplement: Attachment — Submitted filename: Response to Reviewer-1-3-2026-for submission.docx [file pone.0348866.s005.docx]

**Response to Reviewer #3**

We thank the reviewer for the additional evaluation and constructive comments. We have carefully revised the manuscript to address the remaining concerns. Detailed responses are provided below.

**Revision Summary**

We have carefully moderated claims, clarified methodological details, corrected numerical inaccuracies, and strengthened the limitations section. The revised manuscript explicitly:

- Describes all experiments as internally validated,
- Avoids inappropriate cross-study comparisons,
- Frames gene-level classification as proof-of-concept,
- Clearly acknowledges dataset composition limitations.

We appreciate the reviewer’s detailed feedback, which has improved the clarity and rigor of the manuscript.

We address the reviewers’ comments in detail below.

**Major Points**

**1. Was there a held-out test dataset used for Experiments 2 and 3?**

Thank you for raising this important point regarding dataset partitioning. While a separate external dataset was not available for Experiments 2 and 3 due to data scale constraints, we implemented a rigorous validation protocol to ensure generalization.

As detailed in the revised Methods section, all experiments (1-3) were evaluated using strict patient-level five-fold cross-validation. In this framework, each fold functions as a strictly held-out test set for that specific iteration. Crucially, the splitting was performed at the patient level (rather than the image level) to ensure zero data leakage between patients; images from a specific patient never appear in both the training and the held-out test fold simultaneously. Consequently, the reported performance metrics (mean ± SD) reflect the model’s ability to generalize to unseen patient data.

We have now explicitly stated in both the Methods and Discussion sections that all reported results reflect internal cross-validation performance and that no independent external test cohort was used. This limitation is clearly acknowledged.

- Line 208 (highlighted manuscript: Experiment 1: Multi-class classification of IRD versus non-IRD across all patients. This was a binary classification task including all 330 IRD patients and 79 non-IRD patients (mean test set size ~206 studies per fold).
- Experiment 2: Five-class classification task including only IRD patients belonging to Group 1 (ABCA4, NR2E3, RDH12, TRPM1, USH2A). Each patient was assigned to one of the five gene classes. (mean test set size ~60 studies per fold).
- Experiment 3: Five-class classification task including only IRD patients belonging to Group 2 (BEST1, CHM, CNGA3, CRB1, FAM161A). Each patient was assigned to one of the five gene classes. (mean test set size ~39 studies per fold).

**2. What was the data split used for training, validation, and testing for all experiments?**

We have clarified the data partitioning strategy in the Methods section. For each fold:

- 80% of patients were used for training (including internal validation during model optimization),
- 20% of patients were held out for testing,
- The split was performed at the patient level.

This procedure was repeated across five folds, and final metrics were averaged across folds.

This information has now been explicitly described to improve clarity and reproducibility.

Line 225: 2.7 Data partitioning strategy and held-out testing

All experiments were performed using strict patient-level five-fold cross-validation. In each fold, approximately 80% of patients were used for training and 20% were held out for testing. Each fold functions as a strictly held-out test set. Crucially, the splitting was performed at the patient level (rather than the image level) to ensure zero data leakage between patients; images from a specific patient never appear in both the training and the held-out test fold simultaneously.

Within the training set of each fold, a random 10% subset of patients was used as a validation set for early stopping and hyperparameter tuning. This validation set was also separated at the patient level from the remaining training subset.

For Experiments 2 and 3, the test set included only IRD patients belonging to the relevant gene group under evaluation. Each of these experiments constituted a multi-class classification task with five classes corresponding to the five genes in the group. No patients from other genes were included in the test set for these experiments.

This design ensures that model performance reflects true generalization to unseen patients rather than image-level memorization.

No external validation cohorts was available, and all results reflect internal cross-validation performance within a single-center dataset.

**3. It is not clear if for experiments 2 and 3 the test data only included images from patients with one of the 5 genes that are in group 1 or 2 respectively. I.e. were experiments 2 and 3 a classification task with 5 classes in each experiment?**

Yes. Experiments 2 and 3 were structured as five-class classification tasks. For each experiment:

- Only patients carrying one of the five genes defined for that group were included.
- The classification task was restricted to distinguishing among those five genes.
- Cross-validation splits were performed within each defined gene group.

We have clarified this explicitly in the Methods section to avoid ambiguity.

We also emphasize in the revised Discussion that these experiments represent constrained five-class settings and are not directly comparable to large-scale multi-gene classification tasks involving dozens of genes.

Line 225: 2.7 Data partitioning strategy and held-out testing

All experiments were performed using strict patient-level five-fold cross-validation. In each fold, approximately 80% of patients were used for training and 20% were held out for testing. Each fold functions as a strictly held-out test set. Crucially, the splitting was performed at the patient level (rather than the image level) to ensure zero data leakage between patients; images from a specific patient never appear in both the training and the held-out test fold simultaneously.

Within the training set of each fold, a random 10% subset of patients was used as a validation set for early stopping and hyperparameter tuning. This validation set was also separated at the patient level from the remaining training subset.

For Experiments 2 and 3, the test set included only IRD patients belonging to the relevant gene group under evaluation. Each of these experiments constituted a multi-class classification task with five classes corresponding to the five genes in the group. No patients from other genes were included in the test set for these experiments.

This design ensures that model performance reflects true generalization to unseen patients rather than image-level memorization.

No external validation cohorts was available, and all results reflect internal cross-validation performance within a single-center dataset.

**Overall, the authors have overstated their results and interpretation. The manuscript uses previously described methods with nothing new. The unique aspect of this study is the patient population from which the data has been acquired.**

We appreciate the reviewer’s comments regarding overinterpretation. We have carefully revised the Discussion to moderate claims and avoid unintended comparisons.

Line 369: The Eye2Gene study [24,34] represents one of the most comprehensive large-scale efforts to date, including tens of thousands of images and dozens of genes. However, direct numerical comparison between their results and ours should be interpreted with caution. Their task involved classification among a much larger number of genes (up to 63), which is inherently more complex than our five-class experiments. Furthermore, their non-IRD cohort included patients with retinal and/or RPE atrophy from multiple causes, which likely increased the difficulty of binary classification compared to our dataset, where many non-IRD cases had normal or near-normal FAF imaging. Accordingly, our results should be interpreted as a targeted proof-of-concept within a targeted cohort rather than as directly comparable to large-scale multi-gene models. In our targeted five-class settings, we achieved mean accuracies of 0.92 and 0.85 for Group 1 and Group 2, respectively. These results demonstrate strong performance within a limited gene set but should not be interpreted as equivalent to large-scale multi-gene classification tasks.

**Minor points:

Some points in the discussion where the authors have overstated claims or not interpreted results appropriately.**

1. **Line 308, this is incorrect as they also include OCT.**

Thank you for noting this. All references to Eye2Gene results have been substantially modified.

Line 369: The Eye2Gene study [24,34] represents one of the most comprehensive large-scale efforts to date, including tens of thousands of images and dozens of genes. However, direct numerical comparison between their results and ours should be interpreted with caution. Their task involved classification among a much larger number of genes (up to 63), which is inherently more complex than our five-class experiments. Furthermore, their non-IRD cohort included patients with retinal and/or RPE atrophy from multiple causes, which likely increased the difficulty of binary classification compared to our dataset, where many non-IRD cases had normal or near-normal FAF imaging. Accordingly, our results should be interpreted as a targeted proof-of-concept within a targeted cohort rather than as directly comparable to large-scale multi-gene models. In our targeted five-class settings, we achieved mean accuracies of 0.92 and 0.85 for Group 1 and Group 2, respectively. These results demonstrate strong performance within a limited gene set but should not be interpreted as equivalent to large-scale multi-gene classification tasks.

2. **Line 306, the most recent publication predicts from 63 genes rather than 36 for the prior publication.**

We have updated the manuscript to reflect that the more recent publication reports prediction across 63 genes rather than 36.

Line 369: The Eye2Gene study [24,34] represents one of the most comprehensive large-scale efforts to date, including tens of thousands of images and dozens of genes. However, direct numerical comparison between their results and ours should be interpreted with caution. Their task involved classification among a much larger number of genes (up to 63), which is inherently more complex than our five-class experiments. Furthermore, their non-IRD cohort included patients with retinal and/or RPE atrophy from multiple causes, which likely increased the difficulty of binary classification compared to our dataset, where many non-IRD cases had normal or near-normal FAF imaging. Accordingly, our results should be interpreted as a targeted proof-of-concept within a targeted cohort rather than as directly comparable to large-scale multi-gene models. In our targeted five-class settings, we achieved mean accuracies of 0.92 and 0.85 for Group 1 and Group 2, respectively. These results demonstrate strong performance within a limited gene set but should not be interpreted as equivalent to large-scale multi-gene classification tasks.

3. **Line 310, the top 5 accuracy is higher than 76% as reported in the published study.**

All references to Eye2Gene results have been substantially modified.

Line 369: The Eye2Gene study [24,34] represents one of the most comprehensive large-scale efforts to date, including tens of thousands of images and dozens of genes. However, direct numerical comparison between their results and ours should be interpreted with caution. Their task involved classification among a much larger number of genes (up to 63), which is inherently more complex than our five-class experiments. Furthermore, their non-IRD cohort included patients with retinal and/or RPE atrophy from multiple causes, which likely increased the difficulty of binary classification compared to our dataset, where many non-IRD cases had normal or near-normal FAF imaging. Accordingly, our results should be interpreted as a targeted proof-of-concept within a targeted cohort rather than as directly comparable to large-scale multi-gene models. In our targeted five-class settings, we achieved mean accuracies of 0.92 and 0.85 for Group 1 and Group 2, respectively. These results demonstrate strong performance within a limited gene set but should not be interpreted as equivalent to large-scale multi-gene classification tasks.

4. **Line 312, the authors are assuming that the classification task of one in 5 is equivalent to one in 63 (not taking into account averaging for top 5). This is not the same and as this has not been discussed it reads as though they are comparable.**

We agree that classification among five genes is not equivalent to classification among 63 genes, particularly when top-5 accuracy is considered.

In the revised Discussion, we now explicitly state that:

- Five-class experiments are inherently less complex than multi-gene classification tasks involving dozens of genes.
- Direct numerical comparison is inappropriate.
- Our results represent proof-of-concept within a constrained setting.

The text has been modified to prevent any suggestion of equivalence.

Line 437: In addition, the five-class gene grouping strategy simplifies the true clinical scenario in which hundreds of possible genes may be considered. While this approach was necessary due to sample size constraints, it limits direct generalization to broader diagnostic settings.

**5. Line 316 the authors have stated that their results are similar to that described in the recent Eye2Gene paper. The non-IRD cases included in the Eye2Gene paper all had retinal and/or RPE atrophy of different causes whereas the almost all of non-IRD cases in this study will have normal or near normal FAF imaging making the classification task much easier. This has not been noted or discussed.**

We thank the reviewer for this important observation.

We have now explicitly acknowledged in the Discussion that:

- The non-IRD cohort in the referenced large-scale study included patients with retinal and/or RPE atrophy from multiple causes.
- In contrast, many non-IRD cases in our dataset exhibited normal or near-normal FAF imaging.
- This likely made the binary classification task less complex in our study.

This limitation is now clearly discussed.

Line 373: Furthermore, their non-IRD cohort included patients with retinal and/or RPE atrophy from multiple causes, which likely increased the difficulty of binary classification compared to our dataset, where many non-IRD cases had normal or near-normal FAF imaging. Accordingly, our results should be interpreted as a targeted proof-of-concept within a targeted cohort rather than as directly comparable to large-scale multi-gene models.

5. **Line 319-328, the authors do not provide any evidence to substantiate their claims. Which similar models require clinician input and where is the comparator to demonstrate clinical assessment in similar circumstances. The reference from Eye2Gene provided is for assessment of FAF images alone to provide single gene predictions. This is not something clinicians are usually required to perform and the models had more information than single FAF images.**

We agree that the prior wording overstated this point. The revised manuscript:

- Removes unsupported claims regarding comparison with clinician input.
- Avoids implying superiority to human assessment.
- States more conservatively that AI tools may provide decision support, particularly in non-specialist settings.
- Clarifies that prospective evaluation of clinical utility has not been performed.

The Discussion has been substantially moderated in this section.

**Data Availability (Reviewer #3 marked “No”)**

We have reviewed the PLOS Data Policy and ensured compliance. Specifically:

The Data Availability Statement has been revised to ensure transparency and compliance.

Line 124: Due to patient privacy and ethical restrictions, raw retinal images cannot be made publicly available. However, all aggregated performance metrics, confusion matrices, model architecture details, and hyperparameters are provided within the manuscript and supplementary materials. The method will be made available upon acceptance through a public repository in accordance with PLOS data sharing policies.
