# Supplementary material for: Using deep learning to identify inherited retinal diseases based on wide-field retinal imaging data
Source: PLoS One. 2026 May 11;21(5):e0348866. doi: 10.1371/journal.pone.0348866 (PMC13160341; doi:10.1371/journal.pone.0348866)
Supplement: Attachment — Submitted filename: Response to reviewers-15-4-2026.docx [file pone.0348866.s006.docx]

We sincerely thank the reviewer and the editorial board for the careful evaluation of our manuscript and for the positive assessment. We are pleased that the reviewer finds the manuscript to be technically sound and that the previous comments have been adequately addressed. Below, we provide point-by-point responses to the remaining minor comments.

**Comments to the Author**

1. If the authors have adequately addressed your comments raised in a previous round of review and you feel that this manuscript is now acceptable for publication, you may indicate that here to bypass the “Comments to the Author” section, enter your conflict of interest statement in the “Confidential to Editor” section, and submit your "Accept" recommendation.

Reviewer #3: All comments have been addressed

**Response:**
We thank the reviewer for this positive assessment and for the constructive feedback throughout the review process.

2. Is the manuscript technically sound, and do the data support the conclusions?
The manuscript must describe a technically sound piece of scientific research with data that supports the conclusions. Experiments must have been conducted rigorously, with appropriate controls, replication, and sample sizes. The conclusions must be drawn appropriately based on the data presented.

Reviewer #3: Yes

**Response:**
We appreciate the reviewer’s positive evaluation and are grateful for the constructive input, which has helped strengthen the manuscript.

3. Has the statistical analysis been performed appropriately and rigorously?

Reviewer #3: N/A

4. Have the authors made all data underlying the findings in their manuscript fully available?
The [PLOS Data policy](http://track.editorialmanager.com/CL0/http:%2F%2Fwww.plosone.org%2Fstatic%2Fpolicies.action%23sharing/1/010f019d848d2e12-0e51161f-11ad-4f84-a7fe-e1ba1dcd3293-000000/xyNEIlhnzIZlhFpv0AUIRelPegmOxqLKEmj5XJ187U0=258) requires authors to make all data underlying the findings described in their manuscript fully available without restriction, with rare exception (please refer to the Data Availability Statement in the manuscript PDF file). The data should be provided as part of the manuscript or its supporting information, or deposited to a public repository. For example, in addition to summary statistics, the data points behind means, medians and variance measures should be available. If there are restrictions on publicly sharing data—e.g. participant privacy or use of data from a third party—those must be specified.

Reviewer #3: Yes

**Response:**
We thank the reviewer for highlighting the importance of data and code availability. In accordance with PLOS ONE data sharing policies, we have updated the manuscript to include access to the code:

Line 124: Code is available at: https://github.com/timshik/Deep-learning-based-identification-of-inherited-retinal-disease-using-wide-field-retina-imaging.

Line 215: The implemented method is available at link: <https://github.com/timshik/Deep-learning-based-identification-of-inherited-retinal-disease-using-wide-field-retina-imaging>.

5. Is the manuscript presented in an intelligible fashion and written in standard English?
PLOS ONE does not copyedit accepted manuscripts, so the language in submitted articles must be clear, correct, and unambiguous. Any typographical or grammatical errors should be corrected at revision, so please note any specific errors here.

Reviewer #3: Yes

**Response:**
We thank the reviewer for this positive assessment.

6. Review Comments to the Author
Please use the space provided to explain your answers to the questions above. You may also include additional comments for the author, including concerns about dual publication, research ethics, or publication ethics. (Please upload your review as an attachment if it exceeds 20,000 characters)

Reviewer #3: All previous comments have been sufficiently addressed. The authors have acknowledged the value an external dataset would add for testing to support the presented results. Final comments are:
1) please add numbers of patients used (not just samples) for experiments 2 and 3 in methods section lines 202 and 205, respectively.

**Response:**
We thank the reviewer for this helpful suggestion, which improves clarity. We have now explicitly included the number of patients alongside the number of images in the Methods section.

The text has been revised as follows:

Line 202 (Experiment 2):
Experiment 2: Five-class classification task including only IRD patients belonging to Group 1 (ABCA4, NR2E3, RDH12, TRPM1, USH2A). A total of 108 patients (298 images) were included. Each patient was assigned to one of the five gene classes (mean test set size ~60 studies per fold).

Line 205 (Experiment 3):
“Experiment 3: Five-class classification task including only IRD patients belonging to Group 2 (BEST1, CHM, CNGA3, CRB1, FAM161A). A total of 74 patients (196 images) were included. Each patient was assigned to one of the five gene classes (mean test set size ~39 studies per fold).”

2) where relevant in the text, figures and figure legends please use gene names rather than the alphabetised classes representing genes. It is very awkward for the reader to have to check each time which letter represents which gene and would improve readability.

**Response:**
We fully agree with the reviewer that this change improves readability and reduces unnecessary back-and-forth for the reader. We have revised the manuscript accordingly by replacing alphabetized class labels with the corresponding gene names throughout the text, figures, and figure legends wherever applicable
